# Editorial: Rodent-borne diseases: “One Health” perspectives

**DOI:** 10.3389/fvets.2025.1602402

**Published:** 2025-04-22

**Authors:** Veasna Duong, Serge Morand, Philippe Buchy

**Affiliations:** ^1^Institut Pasteur du Cambodge, Pasteur Network, Phnom Penh, Cambodia; ^2^IRL2021 HealthDEEP, Centre National Recherche Scientifique - Kasetsart University - Mahidol University, Bangkok, Thailand; ^3^Faculty of Veterinary Technology, Kasetsart University, Bangkok, Thailand; ^4^Department of Environmental and Social Medicine, Mahidol University, Bangkok, Thailand; ^5^Institut Pasteur du Laos, Pasteur Network, Vientiane, Lao People's Democratic Republic

**Keywords:** rodents, One Health, zoonotic diseases, pet animals, climate change, habitat change, rodent-borne diseases

The One Health approach, as defined by the *One Health High Level Expert Panel (OHHLEP)*, is a collaborative, multisectoral strategy that acknowledges the interdependence of human, animal, and environmental health (1). This holistic perspective is particularly critical in addressing zoonotic diseases, which account for over 60% of emerging infectious diseases worldwide (2). Given their close association with the human environment rodents play a pivotal role in zoonotic disease transmission, necessitating an integrated approach for effective surveillance and control.

Rodents act as reservoirs for a wide array of viral, bacterial, and parasitic pathogens, contributing to both endemic and outbreak-prone diseases. Leptospirosis, caused by *Leptospira* spp., is one of the most widespread rodent-borne zoonotic infections, responsible for an estimated 1.03 million human cases and 58,900 deaths annually (3). Hantaviruses, transmitted through rodent urine and feces, lead to Hemorrhagic Fever with Renal Syndrome (HFRS) and Hantavirus Pulmonary Syndrome (HPS), with mortality rates reaching 40% (4). Arenaviruses from the Old World and New World groups of the genus *Mammarenavirus* are transmitted to humans primarily following contact with the excreta of infected rodents and represent a significant burden of disease with an estimated 100,000 to 300,000 cases of Lassa Fever recorded each year in Africa (5, 6). Plague, caused by *Yersinia pestis*, persists in rodent populations and remains an ongoing public health concern, particularly in Africa, Asia, and South America (7). Rat-bite fever (RBF), a bacterial zoonosis caused by *Streptobacillus moniliformis*, is underreported but can lead to severe septicemia and arthritis if left untreated (8).

Several environmental and anthropogenic factors exacerbate the risk of rodent-borne diseases. Climate change and habitat changes have been linked to alterations in rodent population dynamics and increased pathogen transmission (9). Warmer temperatures and extreme weather events have been associated with hantavirus outbreaks in the Americas and Europe, as changing environmental conditions extend rodent breeding cycles and virus transmission rates (10, 11). Rapid urbanization further exacerbates the problem, as poorly managed waste disposal and inadequate sanitation in densely populated cities facilitate rodent proliferation, increasing the risk of pathogen spillover (12, 13). Additionally, the rise of antimicrobial resistance (AMR) in rodent-associated pathogens has become an emerging concern, with multidrug-resistant *Escherichia coli* and *Salmonella spp*. detected in urban rodent populations, complicating treatment strategies for zoonotic infections (14, 15).

Given these challenges, this Research Topic of *Frontiers in Veterinary Science* adopts a One Health perspective to explore rodent-borne diseases, featuring four pivotal studies that advance our understanding of their clinical implications, ecological dynamics, and surveillance strategies within the One Health framework.

Arpin et al. investigate the role of inter- and transdisciplinary approaches in rodent-borne disease research within an Ecohealth framework. Conducted in a polycrisis era—marked by climate change, biodiversity loss, and global health threats—the study underscores the necessity of integrating ecological, social, and health sciences to develop effective rodent-borne disease mitigation strategies. The authors highlight the challenges of collaborative research, including data-sharing limitations, disciplinary silos, and funding constraints, which hinder comprehensive disease management. They advocate for systems-thinking models and community-based participatory approaches, demonstrating how long-term, integrated strategies can enhance rodent-borne disease surveillance and control. This study underscores the importance of cross-sector partnerships between veterinarians, ecologists, public health experts, and policymakers in developing effective disease prevention efforts.

Building on the theme of clinical challenges, Giraudon et al. present a case report of rat-bite fever (RBF), a neglected zoonotic disease that can cause severe systemic infections. The report describes a severe case of RBF complicated by septic arthritis, emphasizing the diagnostic challenges and the risk of delayed treatment. The case highlights the importance of early recognition and clinical suspicion, especially in patients with rodent exposure or pet rat ownership. Since *S. moniliformis* does not grow well on standard agar media, misdiagnosis is common, leading to delays in treatment. The authors emphasize the need for improved diagnostic tools, clinician awareness, and early antibiotic intervention to prevent life-threatening complications such as endocarditis and septicemia. The study also points to gaps in public health messaging about zoonotic risks associated with pet rodents.

Extending the scope to broader surveillance efforts, Ricardo et al. conduct a systematic review and meta-analysis of the seroprevalence of *Leptospira* in asymptomatic domestic dogs and cats, exploring their potential role as silent reservoirs of infection. Their findings reveal significant *Leptospira* exposure rates among domestic animals, particularly in urban slums and flood-prone regions. These results suggest that dogs and cats may contribute to human leptospirosis transmission cycles, emphasizing the need for routine veterinary screening, vaccination programs, and public awareness campaigns. The study calls for closer collaboration between veterinarians and public health professionals to reduce the spread of leptospirosis.

Shifting the focus to vector-borne pathogens, Wang et al. present a longitudinal surveillance study investigating rodent and tick populations in Zhejiang Province, China. Their research provides critical insights into pathogen circulation and transmission dynamics in rodent hosts, including *Leptospira*, Hantavirus, and *Orientia tsutsugamushi*, with multiple co-infections detected. Notably, Severe Fever with Thrombocytopenia Syndrome Virus (SFTSV) was found in ticks but not in rodents, suggesting a complex ecological interplay between hosts and vectors. The study calls for integrated rodent-vector surveillance programs that incorporate genetic, ecological, and epidemiological data to predict and prevent emerging zoonotic disease threats.

This Research Topic on Rodent-Borne Diseases: One Health Perspectives highlights the critical need for interdisciplinary collaboration, proactive surveillance, and clinical awareness to mitigate rodent-associated health threats. Key takeaways include:

- Strengthening transdisciplinary research to improve zoonotic disease management.
- Integrating veterinary and human medicine for early diagnosis and treatment.
- Addressing factors such as climate, changing landscapes and urbanization that influence rodent-borne disease transmission.
- Enhancing surveillance programs for early pathogen detection and outbreak prevention.

As rodent-borne and other zoonotic diseases continue to pose global health challenges, embracing a One Health approach is essential to mitigate risks and safeguard both human and animal populations.
